# Role of *PRKDC* in cancer initiation, progression, and treatment

**DOI:** 10.1186/s12935-021-02229-8

**Published:** 2021-10-26

**Authors:** Yu Chen, Yi Li, Jiani Xiong, Bin Lan, Xuefeng Wang, Jun Liu, Jing Lin, Zhaodong Fei, Xiaobin Zheng, Chuanben Chen

**Affiliations:** 1 Department of Medical Oncology, Fujian Medical University Cancer Hospital & Fujian Cancer Hospital, Fuzhou, Fujian Province China; 2 Cancer Bio-Immunotherapy Center, Fujian Medical University Cancer Hospital & Fujian Cancer Hospital, Fuzhou, Fujian Province China; 3 Fujian Provincial Key Laboratory of Translational Cancer Medicine, Fuzhou, Fujian Province China; 4 Department of Radiation Oncology, Fujian Medical University Cancer Hospital & Fujian Cancer Hospital, Fuzhou, Fujian Province China; 5 Shanghai Center for Systems Biomedicine Research, Shanghai Jiao Tong University, Shanghai, China; 6 CAS Key Laboratory of Tissue Microenvironment and Tumor, Shanghai Institute of Nutrition and Health, University of Chinese Academy of Sciences, Chinese Academy of Sciences, Shanghai, China; 7 The First Affiliated Hospital of Soochow University and State Key Laboratory of Radiation Medicine and Protection, Institutes for Translational Medicine, Soochow University, Suzhou, Jiangsu China

**Keywords:** *PRKDC*, DNA-dependent protein kinase catalytic subunit, Tumorigenesis, Cancer progression, Cancer treatment

## Abstract

The *PRKDC* gene encodes the DNA-dependent protein kinase catalytic subunit (DNA-PKcs) protein. DNA-PKcs plays an important role in nonhomologous end joining (NHEJ) of DNA double-strand breaks (DSBs) and is also closely related to the establishment of central immune tolerance and the maintenance of chromosome stability. The occurrence and development of different types of tumors and the results of their treatment are also influenced by DNA-PKcs, and it may also predict the results of radiotherapy, chemotherapy, and therapy with immune checkpoint inhibitors (ICIs). Here, we discuss and review the structure and mechanism of action of *PRKDC* and DNA-PKcs and their relationship with cancer.

## Structure of *PRKDC* and DNA-PKcs

**Fig. 1:**
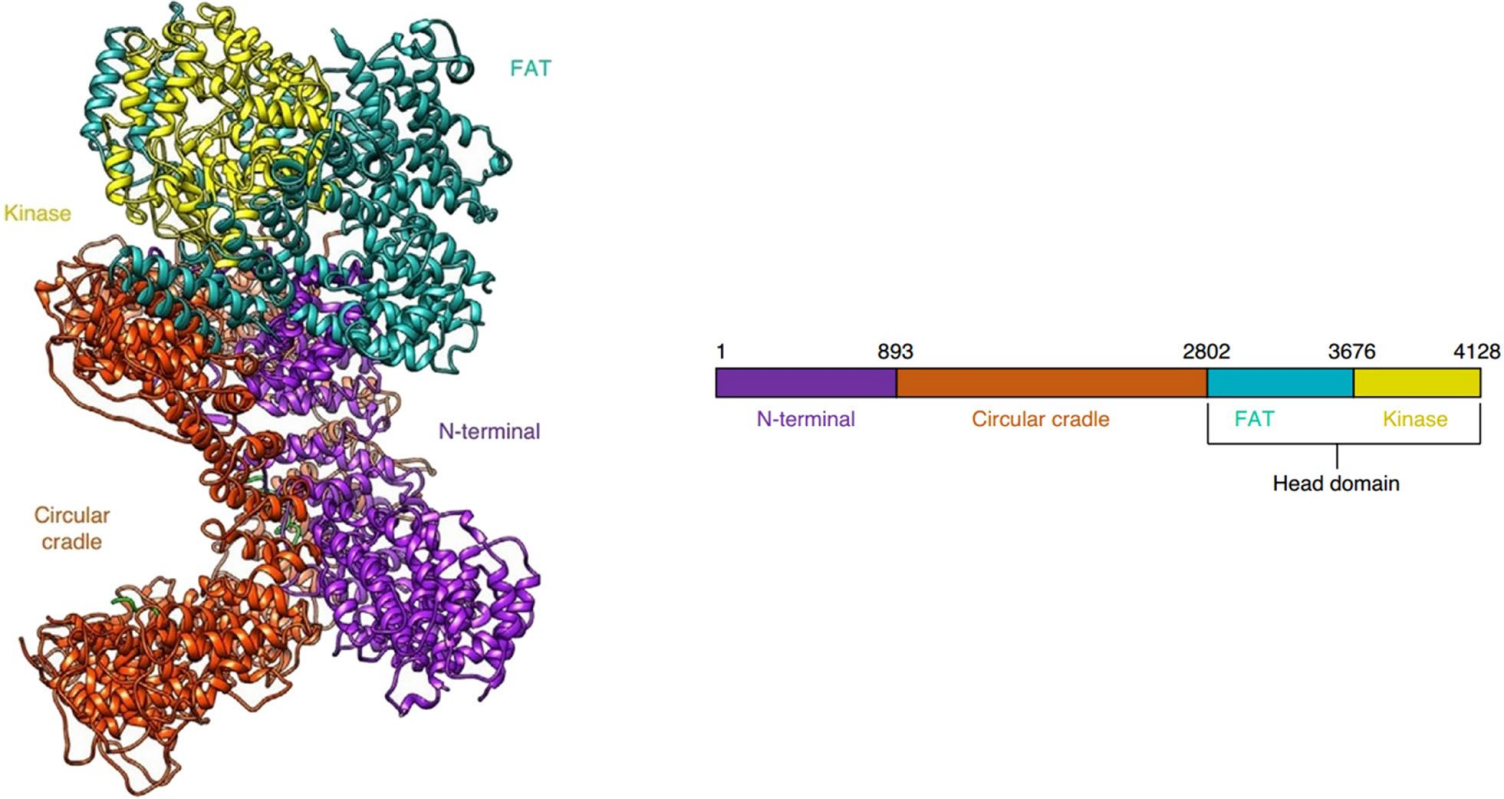
The overall structure of DNA-PKcs (PDB id: 6ZFP). The structural domains are colored according to the schematic shown below the structures. The N terminus is shown in purple, the circular cradle in orange, the FAT domain in teal, and the kinase domain in yellow. (Reference: Chaplin AK, Hardwick SW, Liang S et al. Dimers of DNA-PK create a stage for DNA double-strand break repair. *Nature Structural & Molecular Biology* 2021, 28(1):13–19.)

The *PRKDC* gene (also known as the *XRCC7* gene) is located on chromosome 8q11 and has a transcript length of 12,784 bp. The *PRKDC* gene encodes the DNA-dependent protein kinase catalytic subunit (DNA-PKcs), which is a member of the phosphatidylinositol 3-kinase related kinase (PIKK) protein kinase family and is a serine/threonine protein phosphorylation kinase. The DNA-PKcs protein contains 4128 amino acids; it has a relative molecular weight of 469 kDa and is the highest molecular weight kinase identified thus far. Sibanda et al. [1] analyzed the structure of DNA-PKcs and found that it folded into three large structural units: the N-terminal region (1–892 aa, N1-N4), the Circular Cradle (893–2801 aa, CC-CC5), and the C-terminal Head domain (2802–4128 aa). Existing studies have shown that a central cavity formed by the N-terminal region and the α-helical HEAT repeat sequence of the Circular Cradle is mainly used to mediate the binding to a DNA-Ku70/80 protein dimer complex and provides a passage for the DNA molecule during the DNA repair process [2–4]. The Circular Cradle contains two autophosphorylation clusters, namely the ABCDE cluster and the PQR cluster. The autophosphorylation of the ABCDE cluster promotes the joining of DNA ends. After DNA-PKcs is activated by binding to the DNA-Ku complex, it undergoes autophosphorylation along with phosphorylation of other substrates such as XRCC and H2AX to further complete the double-strand break (DSB) repair process. [1, 5, 6] The C-terminal Head domain contains the FAT domain (2802–3675 aa), the bilobal kinase domain (3676–4100 aa), and the FATC domain (4101–4128 aa). Among these domains, the structure of the bilobal kinase domain is similar to that of the PI3 family, with high basic kinase activity, and its main function is to activate the downstream signaling pathway of PI3K. Previous studies [7, 8] have shown that the kinase activity is not activated by the DNA-Ku protein dimer complex; therefore, this part may not play a major role in DSB repair. The FAT and FATC domains are tightly intertwined with the kinase domain and have a regulatory effect on the catalytic activity of kinases (Fig. 1) [2, 9]. As a catalytic protein, DNA-PKcs together with Ku70 and Ku80, which are two subunits that can bind to DNA and have regulatory function (Ku protein dimers), constitute a DNA-dependent protein kinase (DNA-PK) [10].

## Function and mechanism of* PRKDC* and DNA-PKcs

### *PRKDC* is involved in DNA DSB repair

DNA damage takes many forms, including DNA single-strand breaks (SSBs); DNA DSBs; DNA adducts; and nucleotide mutations, substitutions, deletions, and insertions [11]. Among these forms, DSB is the most cytotoxic form of DNA damage, which is mainly caused by ionizing radiation and radioactive drugs. The mechanisms of repair of DSB include homologous recombination (HR) and nonhomologous end joining (NHEJ); the former requires the same chromosome as the template and can ensure strict pairing to accurately repair DSB, but the repair efficiency is slow. On the other hand, during NHEJ, the two ends of the break are directly joined and repair occurs quickly, which can avoid further serious DNA damage. Although this process cannot ensure accurate repair of DSB, it still plays an important role in maintaining genomic stability [12].

Current results show that DNA-PK is a key protein in NHEJ, and DNA-PKcs encoded by the *PRKDC* gene is the most important component of DNA-PK [12, 13]. When a DSB occurs, the circular Ku70/Ku80 heterodimer recognizes and binds to the broken DNA end to initiate NHEJ, and this complex recruits DNA-PKcs. The DNA-PKcs protein acts as a linker between DNA ends and prevents degradation by exonucleases, a process termed DNA-end tethering [13–16]. DNA-PKcs recruited to the DNA ends is autophosphorylated at the ABCDE cluster [17–19] and subsequently recruits more core proteins of the NHEJ pathway, including XRCC4, XLF, and DNA ligase IV (LIG4) [20–22], which further align and join the DNA ends. As the name implies, this joining process is a direct connection without a template. From the description, it seems to be a very error-prone process, with the possibility of mutation occurrence; however, Betermier et al. [23] showed that NHEJ is an accurate and effective process for joining DNA ends.

An et al. [13] showed that DNA-PKcs can also repair DSBs through other pathways. When ionizing radiation causes DSBs, histone H2AX is phosphorylated to the histone variant H2AX (γH2AX) which plays an important role in the identification and repair of DNA damage; thus, γH2AX is an important marker for DSB repair. The same study [13] also found that DNA-PKcs plays a major role in regulating the phosphorylation of H2AX. The activated DNA-PKcs phosphorylates the 139th serine residue on γH2AX, either directly or indirectly, through the AKT/GSK3β signaling pathway, and repair factors are then recruited to the site of DSBs that coordinate DSB repair.

In the NHEJ process, the recruitment sequence of the various macromolecular proteins involved in DNA repair and the function of the linker are currently being explored; nevertheless, the importance of DNA-PKcs is unquestionable. Experiments conducted by Zhang et al. [24] showed that inhibition of DNA-PKcs directly leads to low DNA repair efficiency, and both in vivo and in vitro experimental results revealed that cells become sensitive to DSBs after exposure to ionizing radiation and cytotoxic drugs. This demonstrates the precise and important role of DNA-PKcs in the NHEJ pathway.

### *PRKDC* involved in the regulation of transcription factors and establishment of central immune tolerance

DNA-PK also interacts with the transcription factor autoimmune regulator (AIRE) to establish central T cell immune tolerance [25, 26]. AIRE is mainly observed in the nucleolus of thymic medullary epithelial cells (MECs) where it plays an important role in the establishment and maintenance of central and peripheral immune tolerance by regulating the heterotopic expression of the tissue-specific antigen (TSA) gene in MECs and inducing the clearance of thymic autoreactive T cells [25, 26]. Abramson et al. [27] used mass spectrometry to identify a group of AIRE-related proteins, and the results showed that DNA-PK exhibited the highest correlation with AIRE. Liiv et al. [28] showed that DNA-PK can phosphorylate Thr68 and Ser156 of AIRE in vitro, thereby allowing AIRE to exert its regulatory function. In cells lacking DNA-PKcs, AIRE loses its ability to bind to DNA and activate transcription of the TSA genes; this finding indicates that DNA-PKcs can guide AIRE to the transcriptional activation site. Therefore, DNA-PKcs is one of the important cofactors for AIRE to perform its functions. DNA-PKcs interacts with AIRE to promote the transcription of TSAs and the subsequent elimination of autoreactive T cells. The abnormal function of DNA-PKcs results in the development of inflammatory disease with organ-specific autoimmunity. This also suggests an important role of DNA-PK in maintaining AIRE-dependent autoimmune tolerance [29].

### *PRKDC* is involved in the maintenance of chromosome stability

Telomeres are structures at the ends of chromosomes that contain guanosine repeats. Their sequence and structure form a “cap” that protects telomeres from degradation. Therefore, telomeres play an important role in genome stability and prevention of the development of malignant cells. Current studies [30, 31] have shown that DNA-PK plays an important role in maintaining telomere length and stability. DNA-PK defects can accelerate telomere degradation and cell apoptosis, leading to increased chromosomal instability and tumorigenesis. DNA-PKcs is also a key regulator of cell mitosis. Lee et al. [32] showed that DNA-PKcs plays an important role in the formation of spindles and in the attachment of microtubules to chromosomes during mitosis. The depletion or functional inhibition of DNA-PKcs leads to chromosome dislocation and mitotic dysfunction, which further increases chromosomal instability. Shang et al. [33] also found that the inactivation of DNA-PKcs led to spindle destruction and mitotic catastrophe.

### The effect of *PRKDC* on cell apoptosis

**Fig. 2:**
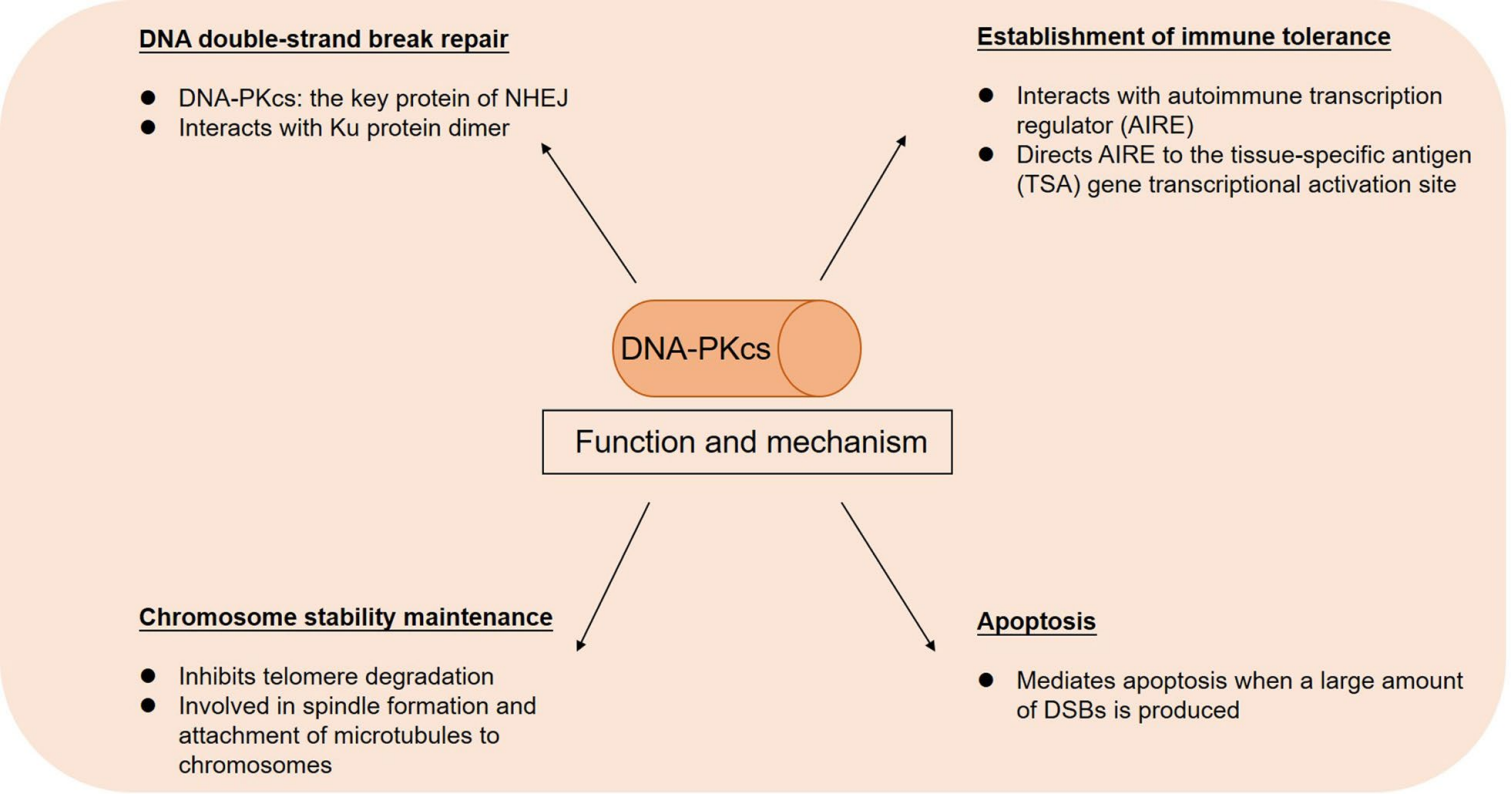
Function and mechanism of DNA-PKcs

As mentioned above, when DSBs occur, DNA-PKcs and Ku70/80 work together to repair DNA damage. When a large amount of DSBs is produced in the cell, the DNA-PK complex also activates the apoptosis program. Abe et al. [34] showed that when high-dose etoposide induces a large amount of DSBs and the activated DNA-PK exceeds the level required for DNA damage repair (DDR), the excess fraction leads to phosphorylation of the Artemis protein which mediates apoptosis. Other studies have also shown that DNA-PK may activate the apoptosis program in ionizing radiation-exposed cells by phosphorylating the p53 protein (Fig. 2) [35, 36].

## Relationship between *PRKDC* and tumor

### *PRKDC* mutation rate in tumors

The mutation rate of *PRKDC* and the expression level of DNA-PKcs in various tumors are quite different. Chen et al. [37] conducted statistical analysis on the mutation rate of *PRKDC* as reported in the Cancer Genome Atlas (TCGA) and the Chinese population database; the authors found that *PRKDC* had a high mutation rate in several tumors, such as colorectal cancer, gastric cancer, and endometrial cancer, with a high correlation with microsatellite instability. In the TCGA database, *PRKDC* mutations were found in 51 (9.66%) of 528 colorectal cancers, 42 (9.63%) of 436 gastric cancers, and 23 (9.27%) of 248 endometrial cancers. In contrast, *PRKDC* mutations were less common in some other types of tumors such as thyroid cancer (0.99%), glioblastoma (1.37%), and liver cancer (1.61%).

### DNA-PKcs expression levels are different in different types of tumors

Many studies have analyzed the expression levels of DNA-PKcs in clinical samples of various types of tumors. On the one hand, these results are expected to explain some association between DNA-PKcs activity and the occurrence and development of cancer, and on the other hand, they provide some important information for predicting the tumor response to radiotherapy and chemotherapy.

In most types of tumors, the expression of DNA-PKcs in tumor tissues was found to be significantly higher than that in adjacent tissues. Lu et al. [38] extracted and quantified the total protein of 16 fresh colorectal cancer surgical specimens and normal adjacent tissues, and both immunohistochemistry (IHC) and western blot results showed that the expression of DNA-PKcs in colorectal cancer specimens was significantly higher than that in adjacent tissues (P < 0.05). Zhang et al. [39] analyzed 80 pairs of gastric cancer tissues and adjacent tissues and found that *PRKDC* was overexpressed in most subtypes of gastric cancer tissues and that *PRKDC* was the most significantly upregulated gene related to DDR. Maag et al. [40] collected samples from 17 patients with normal squamous esophagus, 22 patients with Barrett’s esophagus, and 12 patients with esophageal adenocarcinoma for immunohistochemical analysis of DNA-PKcs expression. The IHC density score showed that the expression of DNA-PKcs in esophageal adenocarcinoma tissues was significantly higher than that in Barrett’s esophagus and normal squamous esophagus tissues. A similar expression pattern was also found in renal cell carcinoma (RCC), nasopharyngeal carcinoma, and non-small cell lung cancer (NSCLC) [41–44].

Other types of tumors have shown reduced or lack of DNA-PKcs expression. For example, it was found that the expression of DNA-PKcs was decreased or absent in ovarian cancer tissues as compared to that in normal tissues. In the included 20 normal ovarian tissues, 20 benign ovarian tumors, 20 borderline ovarian tumors, and 40 malignant ovarian tumors, the positive rate of DNA-PKcs in each group was 100%, 95%, 90%, and 60%, respectively [45]. Lee et al. [46] also used IHC to analyze the expression of DNA-PKcs in 279 patients with gastric cancer, and they found that DNA-PKcs expression was negative in 23% of gastric cancer samples and that the lack of DNA-PKcs was associated with lymph node metastasis and a poor prognosis.

The difference (increase or decrease) in the expression level of DNA-PKcs in tumor tissues as compared to that in adjacent normal tissues suggests that DNA-PKcs may play a role in the process of tumorigenesis and cancer development.

### The role of PRKDC in the process of tumorigenesis and cancer development

By analyzing the differential expression of DNA-PKcs in tumor tissues and normal tissues, many studies [38, 42, 47–55] have further shown that the expression level of DNA-PKcs is also closely related to the genesis, development, invasion, and distant metastasis of various types of tumors.

**Fig. 3:**
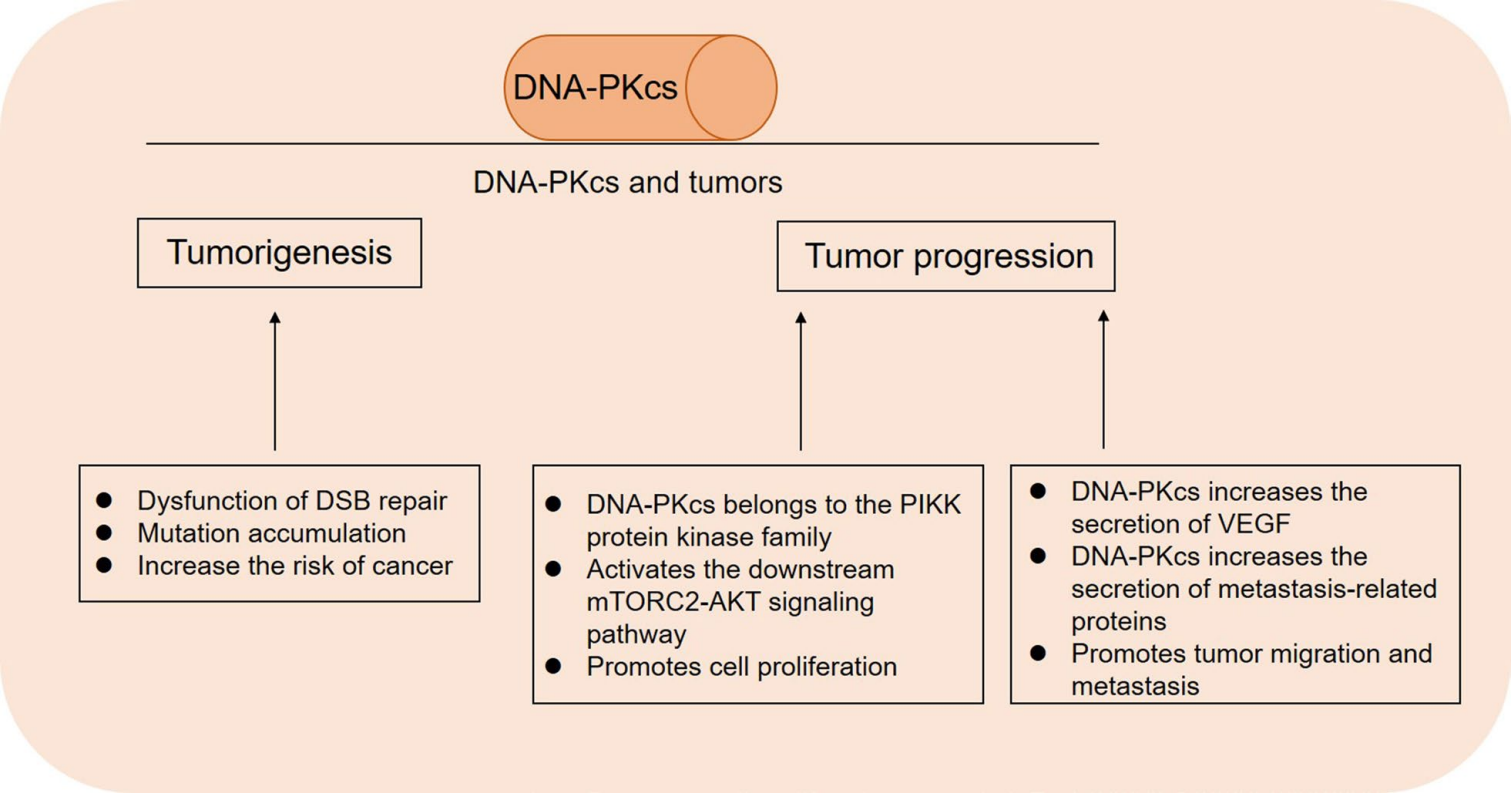
DNA-PKcs and tumorigenesis and tumor progression

The recognized effects and possible underlying mechanisms of *PRKDC* and DNA-PKcs in tumorigenesis and cancer development can be summarized from the following findings. First, *PRKDC* mutations and DNA-PKcs expression defects cause dysfunction of DSB repair. This leads to genome instability and accumulation of mutations that increase the risk of cancer. It has been found that tumorigenesis in many types of cancers are related to DNA-PKcs defects, and its impaired activity makes the human body prone to the development of various malignant tumors. McKean-Cowdin et al. [47] proposed that co-mutations in DDR genes were associated with an increased risk of glioblastoma multiforme (GBM), and a variant of *PRKDC* increased the risk of glioma by 44%. The 2004 study by Wang et al. [48] also showed that another variant of *PRKDC* increased the risk of glioma by 1.82-fold. Teneng et al. [49] demonstrated through an in vitro study that the knockdown of DNA-PKcs significantly increased the amount of detectable DNA damage after bleomycin treatment and led to malignant transformation of bronchial epithelial cells and tumorigenesis. Second, DNA-PKcs affects tumor susceptibility and progression by regulating certain tumor suppressor genes and signaling pathways. Mori et al. [50] found that DNA-PKcs is a candidate regulatory factor for the radiation-induced apoptosis 1 (*Rapop1*) gene, which affects the susceptibility to radiation-induced lymphoma. As mentioned above, DNA-PKcs is a member of the PIKK protein kinase family; thus, it can act as an upstream regulator to activate mTORC2-AKT, a cell proliferation-related signaling pathway. Zheng et al. [41] found that in RCC, the overexpression of DNA-PKcs was significantly associated with the activation of the mTORC2-AKT signaling pathway. The knockdown of DNA-PKcs inhibited AKT phosphorylation, and the expression of *HIF-2α* (an mTORC2 regulatory gene) was also significantly downregulated. Third, the DNA-PKcs protein also participates in tumor progression by changing the tumor microenvironment. Kotula et al. [51] inoculated DNA-PKcs-deficient human melanoma cells into nude mice to generate subcutaneous tumors. They found that these tumors had significantly fewer blood vessels than non-DNA-PKcs-deficient tumors, and the proliferation index was low. The survival analysis of mice showed that the distant metastasis-free survival (MFS) of DNA-PKcs-deficient mice was significantly better than that of non-DNA-PKcs-deficient animals. Liu et al. [52] also suggested that inhibition of DNA-PKcs in glioblastoma could reduce the secretion of VEGF, thus inhibiting the migration and metastasis of tumors. Kotula et al. [51] also reported that DNA-PKcs can control a variety of metastasis-related proteins, including matrix metalloproteinases, to affect the tumor microenvironment and promote tumor migration. In other malignancies, including colorectal cancer, gastric cancer, hepatocellular carcinoma, and nasopharyngeal carcinoma, elevated DNA-PKcs protein levels were found to be significantly associated with lymphatic and distant metastasis in the late stage of tumor growth as well as with low differentiation of tumor cells (Fig. 3) [38, 42, 53–55].

Both *PRKDC* and DNA-PKcs influence genesis, development, invasion, and metastasis of tumors through various mechanisms, and further prognostic analysis also suggests that DNA-PKcs expression can help to predict the prognosis of patients. After studying the influence of the DNA-PKcs expression level on the clinical staging and lymphatic and distant metastasis of colorectal cancer, Lu et al. [38] further proved that the DNA-PKcs expression level was negatively correlated with the 5-year survival rate of patients. Xing et al. [44] analyzed the gene expression profile of NSCLC cells, and the results showed that patients with high DNA-PKcs or ATM expression levels in tumor sample/normal tissue sample (T/N) ratio had a significantly increased risk of death. In other cancers such as gastric cancer, liver cancer, breast cancer, and nasopharyngeal carcinoma, the expression level of DNA-PKcs is also negatively correlated with prognosis [39, 42, 53, 56].

In addition to DNA-PKcs expression in tumor cells, its expression level in peripheral blood lymphocytes (PBLs) of tumor patients is also related to the biological behavior of tumors and patient prognosis. At present, it is generally believed that the decreased expression of DNA-PKcs in PBLs is associated with malignant progression and a poor prognosis for patients. Someya et al. [57] found that the activity of DNA-PKcs in PBLs was generally decreased in patients with advanced cancer, which was correlated with an invasive phenotype and a poor prognosis. Auckland et al. [58] reported that the decrease in DNA-PKcs in peripheral monocytes was associated with the development of lung cancer.

It was also found that in patients with cervical cancer and breast cancer, the DNA-PKcs expression level in PBL was significantly lower than that in healthy volunteers, and the reduction in DNA-PKcs expression was associated with an increased risk of cancer [59].

## *PRKDC* and tumor treatment

### *PRKDC* and response to chemotherapy and radiotherapy

#### Association of DNA-PKcs expression with response to radiotherapy and chemotherapy

Radiotherapy and chemotherapy are very important in cancer treatment, and their anticancer mechanisms are related to the induction of lethal DSBs in tumor cells [60]. As a key protein in NHEJ, DNA-PKcs plays an important role in repairing DSBs; therefore, *PRKDC* mutations or abnormal expression of DNA-PKcs is associated with the response to radiotherapy and chemotherapy. An increased level of DNA-PKcs enhances the repair of DSBs in cells that weakens the lethal effect of radiotherapy and cytotoxic drugs, thereby making tumors more resistant to radiotherapy and chemotherapy. Sun et al. [61] found a significant correlation between DNA-PKcs expression and the response to chemotherapy in patients with breast cancer. The expression level of DNA-PKcs in tumor cells of chemotherapy-resistant patients was significantly higher than that in tumor cells of patients who responded to chemotherapy. In addition, among the patients who received chemotherapy, the overall survival of those with high DNA-PKcs expression levels was significantly reduced compared to that of patients with low DNA-PKcs expression levels. Previous studies have also used *PRKDC* knockout breast cancer cell lines and untreated breast cancer cell lines to inoculate mice. In vivo experiments confirmed that the expression level of DNA-PKcs directly affects the tumor response to chemotherapy, and *PRKDC* knockout can sensitize the inoculated tumors to chemotherapy with significant tumor regression. Shao et al. [62] also found that the DNA-PK expression level in glioma cells had a high positive correlation with sensitivity to cisplatin in vitro, and the expression of DNA-PK was high in patients who did not respond to chemotherapy. Studies on oral squamous cell carcinoma and cervical squamous cell carcinoma also showed that the expression of DNA-PKcs was upregulated in surviving tumor cells after radiotherapy, which indicated that these cells were resistant to radiotherapy and survived selectively [63, 64].

#### Application of DNA-PKcs inhibitors in tumor chemoradiotherapy

The upregulation of DNA-PKcs leads to radiotherapy and chemotherapy resistance. Conversely, inhibition of DNA-PKcs can induce the programmed cell death of tumors and enhance the cell-killing effect of chemotherapy and radiotherapy by inhibiting the repair of DSBs. It has been confirmed that a variety of small molecules can inhibit the activity of DNA-PKcs, including NU7441, NU7026, SU11752, and IC87361. Attempts have been made to combine DNA-PKcs inhibitors with chemoradiotherapy or other antitumor agents for tumor treatment. Sarah et al. [65] showed that the combination of mitoxantrone and the DNA-PKcs inhibitor NU7441 can improve the treatment response of patients with high-risk chronic lymphocytic leukemia (CLL). Wojciech et al. [66] reported that NU7441 can increase the sensitivity of three types of breast cancer cell lines to radiation and adriamycin. Cindy et al. [67] showed that the phosphorylation of DNA-PKcs can accelerate the repair of damaged DNA in cells induced by ionizing radiation, while DNA-PKcs inhibitors can inhibit this process. In vivo experiments showed that the survival rate of mice treated with radiotherapy combined with a DNA-PKcs inhibitor was significantly higher than that of mice treated with radiotherapy alone. Zhang et al. [24] revealed that treatment of radiation-resistant cells with NU7441 can re-sensitize the cells to radiotherapy both in vivo and in vitro.

DNA-PKcs is also a protein kinase necessary for cell cycle progression during chromosome separation and mitosis, and the cell cycle checkpoint arrest caused by inhibiting DNA-PKcs can also lead to increased apoptosis and enhanced cell killing by radiotherapy and chemotherapy. Azad et al. [68] reported that in NSCLC cells, the inhibition of DNA-PKcs can arrest the irradiated cells in the G2/M phase and accelerate apoptosis.

Other studies have shown that the inhibition of DNA-PKcs or a decrease in DNA-PKcs expression levels can also activate autophagy in irradiated cells, thereby contributing to the efficacy of radiotherapy. Zhuang et al. [69] showed that in irradiated glioma cells, inhibition of DNA-PKcs promotes autophagy of tumor cells by inducing the expression of two autophagy markers, namely microtubule-associated protein light chain 3 (LC3) and Beclin1, and it increases the death of irradiated cells.

### *PRKDC* and immunotherapy

Immunotherapies have received increasing attention in recent years, and among the most important ones are the immune checkpoint inhibitor (ICI) therapies [70, 71]. Currently, an important factor in predicting the response to ICIs is tumor mutation burden (TMB) [37], which refers to the number of nonsynonymous mutations per million bases of the tumor cell genome. The damage to the DDR system is closely related to the TMB, and a functional defect of the DDR system leads to increased genomic instability and accumulation of mutations in cells [37]. *PRKDC* is a key gene in the NHEJ pathway and in DSB repair, and its relationship with immunotherapy is being gradually explored. Several current studies have shown that mutations in the *PRKDC* gene are associated with a better response to ICIs, mainly due to the increased TMB of *PRKDC* mutant cells and the phenotype of immune cell infiltration. Rizvi et al. [72] performed whole-genome sequencing on 34 cases of NSCLC where patients were treated with pembrolizumab to detect genomic biomarkers that can predict the response to PD-1 monoclonal antibody treatment. They showed that, of the 14 patients with a durable clinical benefit (DCB), two had *PRKDC* mutations, while out of 20 patients with nondurable clinical benefit (NDCB), none of them had *PRKDC* mutations. Chen et al. [37] comprehensively analyzed the relationship between *PRKDC* mutations and TMB, tumor microenvironment, and treatment outcomes of pan-cancer patients after ICI therapy. They found that *PRKDC* mutations were significantly correlated with elevated TMB and an inflammatory tumor microenvironment. In the clinical cohorts of NSCLC and melanoma patients treated with ICIs, they reported that *PRKDC* mutations are confirmed to be associated with a better prognosis. Tan et al. [73] analyzed previous immunotherapy-related studies and found that, in melanoma, NSCLC, and kidney cancer, the ICI response rates of patients with *PRKDC* mutations reached 53.8%, 75%, and 50%, respectively (Table 1). Moreover, the CT26 animal model was also used in this study to confirm that the *PRKDC* knockout cells had a better response to ICIs as shown by a significant tumor volume reduction.

**Table 1:** Response rate and prevalence of *PRKDC* mutations in data cohorts from several clinical cohorts [69]

Paper	Cancer	Sample size	Sample with PRKDC mutation	Responder with PRKDC	Response rate (%)
Synder et al.	Melanoma	64	2 (3.1%)	2	100
Rizvi et al.	NSCLC	31	3 (9.7%)	2	66.70
Van Allen et al.	Melanoma	110	9 (8.2%)	4	55.60
Anagnostou et al.	NSCLC	4	1 (25%)	1	100
Maio et al.	Renal cell carcinoma	98	2 (2.0%)	1	50
Riaz et al.	Melanoma	65	2 (3.1%)	1	50

## Conclusion

Current evidence shows that *PRKDC* and its encoded protein DNA-PKcs perform a variety of important functions in cells, including DNA DSB repair, participation in the establishment of immune tolerance, and maintenance of chromosome and genome stability. Changes in the expression level of DNA-PKcs also affect the occurrence and development of tumors. Regarding tumorigenesis, DNA-PKcs defects lead to genome instability and increased tumor susceptibility. In the process of tumor development, the enhanced activity of DNA-PKcs affects the regulation of some tumor suppressor genes, leading to malignant progression characterized by poor differentiation and increased aggressiveness. Regarding tumor treatment, the elevated DNA-PKcs levels will enhance the resistance of cancer cells to DNA damage and chemoradiotherapy, thereby leading to a poor prognosis for patients. Moreover, several studies have shown that *PRKDC* mutations are associated with a higher TMB and a better response to ICIs; thus, *PRKDC* is also expected to become another predictive biomarker for ICI therapy.

Future research studies need to consider the following aspects to improve outcome: (1) several key amino acid positions in the domains of the DNA-PKcs protein are particularly important for DSB repair, such as S2023, S2029, S2041, S2053, and S2056 [1], and there is still a lack of research on the effects of mutations at these sites on protein function; and (2) because the main mechanism of the association between DNA-PKcs and tumor therapy (radiotherapy, chemotherapy, and immunotherapy) is based on the integrity or defect of the former’s DSB repair function, the exploration of key site mutations in DNA-PKcs can help to identify biomarkers for chemoradiotherapy and immunotherapy and more accurately predict therapeutic outcomes.

### Search strategy and selection criteria

Data for this review were obtained by searching PubMed and references from relevant articles using the search terms “DNA-PKcs” and “PRKDC”. Articles published in English between 1992 and 2020 were included in this review.

## Data Availability

Data for this Review were identified by searches of PubMed.
